# Clonally Expanded Decidual Effector Regulatory T Cells Increase in Late Gestation of Normal Pregnancy, but Not in Preeclampsia, in Humans

**DOI:** 10.3389/fimmu.2018.01934

**Published:** 2018-08-24

**Authors:** Sayaka Tsuda, Xiaoxin Zhang, Hiroshi Hamana, Tomoko Shima, Akemi Ushijima, Kei Tsuda, Atsushi Muraguchi, Hiroyuki Kishi, Shigeru Saito

**Affiliations:** ^1^Department of Obstetrics and Gynecology, University of Toyama, Toyama, Japan; ^2^Department of Innovative Cancer Immunotherapy, Graduate School of Medicine and Pharmaceutical Sciences (Medicine), University of Toyama, Toyama, Japan; ^3^Department of Immunology, Graduate School of Medicine and Pharmaceutical Sciences (Medicine), University of Toyama, Toyama, Japan

**Keywords:** effector regulatory T cell, human pregnancy, miscarriage, preeclampsia, regulatory T cell, T cell repertoire

## Abstract

**Background:** Regulatory T (Treg) cells are necessary for the maintenance of allogenic pregnancy. However, the repertoire of effector Treg cells at the feto-maternal interface in human pregnancy remains unknown. Our objective was to study T cell receptor (TCR) repertoires of Treg cells during pregnancy compared to normal and complicated pregnancies.

**Methods:**Paired samples of peripheral blood and decidua in induced abortion and miscarriage cases were obtained from consenting patients. CD4^+^CD25^+^CD127^low/−^CD45RA^−^ effector Treg cells were single-cell sorted from mononuclear cells. cDNAs of complementarity determining region 3 (CDR3) in TCRβ were amplified from the single cells by RT-PCR and the sequences were analyzed. The TCRβ repertoires were determined by amino acid and nucleotide sequences. Treg cells were classified into clonally expanded and non-expanded populations by CDR3 sequences.

**Results:** We enrolled nine induced abortion cases in the 1st trimester, 12 cases delivered without complications in the 3rd trimester, 11 miscarriages with abnormal chromosomal karyotyped embryo, seven miscarriages with normal chromosomal karyotyped embryo, and seven cases of preeclampsia [median gestational week (interquartile range): 7 (7–9), 39 (38–40), 9 (8–10), 8 (8–10), and 34 (32–37), respectively]. The frequency of clonally expanded populations of effector Treg cells increased in decidua of 3rd trimester cases compared to 1st trimester cases [4.5% (1.4–10.8%) vs. 20.9% (15.4–28.1%), *p* < 0.001]. Clonally expanded Treg cells were rarely seen in peripheral blood. The ratio of clonally expanded populations of decidual effector Treg cells in miscarriages with abnormal and normal embryos was not significantly different compared with that in 1st trimester normal pregnancy. Interestingly, clonally expanded populations of effector Treg cells decreased in preeclampsia compared with that in 3rd trimester normal pregnancy [9.3% (4.4–14.5%) vs. 20.9% (15.4–28.1%), *p* = 0.003]. When repertoires in previous pregnancy and subsequent pregnancy were compared, some portions of the repertoire were shared.

**Conclusion:** TCR repertoires of decidual effector Treg cells are skewed in the 3rd trimester of normal pregnancy. Failure of clonal expansion of populations of decidual effector Treg cells might be related to the development of preeclampsia.

## Introduction

Regulatory T (Treg) cells are important in maintaining feto-maternal tolerance during pregnancy in humans and mice (1–6). Previous studies demonstrated the existence of fetal antigen specific Treg cells in a murine model of pregnancy (7–9). Rowe et al. (9) demonstrated that memory type-fetal antigen specific Treg cells induced fetal antigen specific tolerance in second pregnancy in mice. These results may explain why first pregnancy is a risk factor for preeclampsia. We have reported that fetal antigen specific Treg cells are recruited to uterine draining lymph nodes just before implantation in mice (10). Tilburgs et al. (11) suggested that human decidual Treg cells recognize self-fetal antigens by mixed lymphocyte reaction against umbilical cord blood. A suppressive reaction by decidual Treg cells, but not by systemic Treg cells, has been described (11). These findings suggest that fetal antigen specific memory type Treg cells induce feto-maternal tolerance at the feto-maternal interface in both mice and humans, although fetal antigen specific Treg cells have not yet been identified as a T cell receptor (TCR) repertoire in humans.

Human CD4^+^FoxP3^+^ cells contain a CD4^+^CD45RA^−^FoxP3^high^ effector/activated Treg cell subset, CD4^+^CD45RA^+^FoxP3^low^ naïve/resting Treg cell subset, and CD45^+^CD45RA^−^FoxP3^low^ effector T cell subset (12, 13). CD4^+^CD45RA^−^FoxP3^high^ effector Treg cells have the highest suppressive capability among these subsets. During human late gestation, CD4^+^CD45RA^−^FoxP3^high^ effector Treg cells are the dominant Treg cell subset in peripheral blood and decidua (14). We have shown that CD4^+^CD45RA^−^FoxP3^high^ effector Treg cells are significantly decreased in decidua of cases of miscarriage with normal chromosomal karyotyped embryo (15). Thus, the effector Treg cell subset might contain fetal antigen specific populations in human.

Previous reports suggested that systemic and local maldistribution and dysfunction of Treg cells could be one of the etiologies of miscarriage and preeclampsia (16–21). T cell receptor β variable (TRBV) repertoires of total Treg cells in peripheral blood and decidua were not significantly different between preeclampsia and normal pregnancy (22). Thus, how Treg cells relate to the development of preeclampsia remains unknown.

We hypothesized that decidual effector Treg cells that recognize fetal antigens are clonally expanded at the feto-maternal interface. To study the clonality of effector Treg cells, we used a single-cell based TCR repertoire analysis method that we previously described (23, 24). We also aimed to show whether altered TCR repertoires of effector Treg cells are present in miscarriage or preeclampsia.

## Materials and methods

### Subjects

The enrolled cases included nine cases of artificial abortion in the 1st trimester, 11 cases of 1st trimester miscarriage with abnormal embryo karyotype, seven cases of 1st trimester miscarriage with normal embryo karyotype, 12 cases delivered without pregnancy complications in the 3rd trimester, and seven cases delivered in the 3rd trimester with preeclampsia. Written informed consent was obtained from all the patients in accordance with a protocol approved by the Ethical Review Board of University of Toyama. Fetal heartbeat was confirmed before artificial abortion (induced abortion in the 1st trimester of normal pregnancy). Miscarriage was diagnosed when the fetal heart beat was lost, or when the fetal heartbeat was not detected inside the gestational sac for more than 2 weeks. All artificial abortion and miscarriage treatments were performed by dilation and curettage. Fetal chromosomal karyotype was determined by G-band staining in miscarriage cases. Preeclampsia was diagnosed when blood pressure exceeded 140/90 mmHg and urinary protein exceeded 0.3 g per day after the 20th week of gestation (25). Ten milliliters of venous blood and decidual sample were obtained simultaneously when induced abortion was performed or when subjects delivered a baby. The patients were recruited at Toyama University Hospital, Otogi no Mori Lady's Clinic and Yoshie Ladies Clinic.

### Mononuclear cell isolation

Peripheral blood mononuclear cells (PBMC) were isolated by Ficoll Hypaque (Lymphoprep™; Alere Technologies, Norway) density gradient centrifugation. First trimester decidua was isolated from uterine content that was collected by induced abortion. Third trimester decidua was dissected from maternal surface of the delivered placenta. Decidua was rinsed with phosphate buffered saline (PBS) until the blood was removed, minced with a pair of scissors to produce 1–2 mm pieces, and filtered through 32-μm nylon mesh. All samples were cryopreserved.

### Single-cell sorting

**Figure 1:**
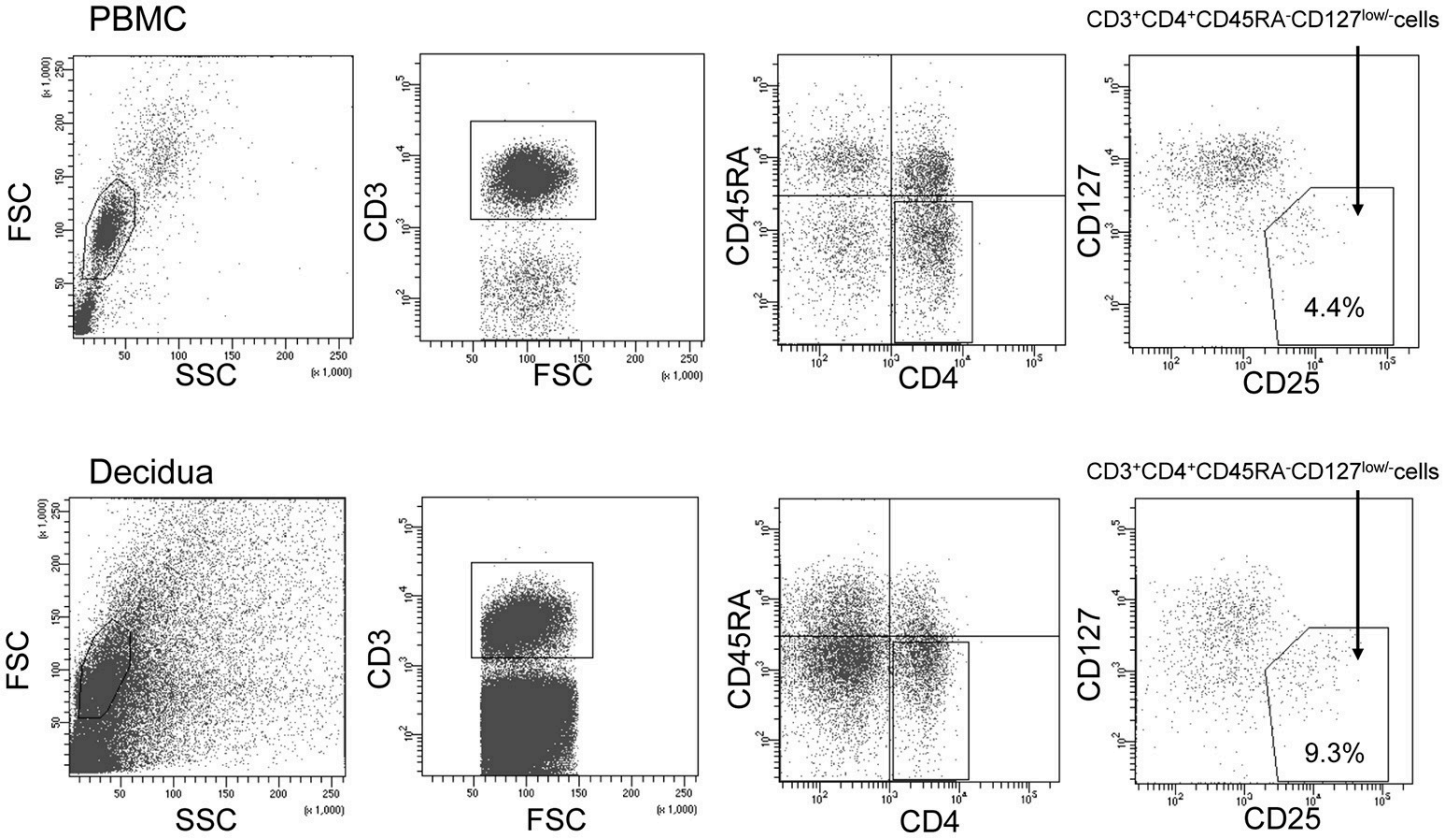
Gating strategy to obtain CD4^+^CD45RA^−^CD25^+^CD127^low/−^effector Treg cells. Lymphocyte in the peripheral blood (upper column) and decidua (lower column) were gated on forward and side scatter parameters. CD3^+^CD4^+^ T cells were classified into CD45RA^+^ naïve T cells and CD45RA^−^ effector T cells. Among CD45RA^−^ effector T cells, CD25^+^CD127^low/−^ effector Treg cells were single-cell sorted.

To sort the effector Treg cells, the following monoclonal antibodies were used: anti-CD3 (APC; BD Bioscience, USA), anti-CD4 (PerCP cy5.5; BD Bioscience), anti-CD45RA (APC cy7; BioLegend, USA), anti-CD25 (PE cy7; BioLegend), and anti-CD127 (PE; BD Bioscience). PBMC and decidual mononuclear cells were stained by these antibodies for 20 min on ice. After staining, the cells were washed with PBS and analyzed using a FACSAria II flow cytometer (BD Biosciences). CD3^+^CD4^+^CD45RA^−^CD25^+^CD127^low/−^ effector Treg cells were single-cell sorted into wells of a 96-well PCR plate. The gating strategy used to sort the effector Treg cells is presented in Figure 1.

### TCR repertoire analysis of effector treg cells by single-cell RT-PCR and sequencing

TCRs and FoxP3 cDNAs were amplified from single cells using one-step multiplex RT-PCR as described previously (24). All PCR primers are listed in Supplementary Table 1. Contents of the PCR reaction mixture are listed in Supplementary Table 2. Five microliters of the RT-PCR mixture was added to each well containing a single effector Treg cell. One-step RT-PCR was performed with the following program: 40 min at 45°C for the RT reaction, 98°C for 1 min and 30 cycles of 98°C for 10 s, 52°C for 5 s, and 72°C for 1 min. The products were diluted 10-fold and 2 μL of each was added to 18 μL of the second PCR mixture. In the second cycle, TCRβ and FoxP3 cDNAs were amplified. The program for the second PCR was as follows: 98°C for 1 min and 35 cycles of 98°C for 10 s, 52°C for 5 s, and 72°C for 30 s. The second PCR products were used for direct sequencing to determine CDR3 of TCRβ. The TCR repertoire was analyzed with the IMGT/VQuest tool (http://www.imgt.org/). We classified effector Treg cells with identical CDR3 as the clonal population and those with unique CDR3 as the non-clonal population. To compare the clonality of the effector Treg cells, frequencies of clonal populations among the analyzed TCRs were calculated. For the assessment of TCR repertoire distribution, we calculated the Gini-coefficient as previously described (26). This coefficient was originally used in economic studies to describe income distribution. However, it is also useful to describe TCR repertoire distribution (26, 27). The Gini coefficient (*G*) is calculated as:

$$G=\sum_{i\;=\;1}^{n}\left(2i-n-1\right)xi/n\sum_{i\;=\;1}^{n}xi$$

where “*xi*” indicates the abundance of the *i*^th^ sequence and “*n*” indicates the total number of TCR sequences. The score ranges from 0 to 1; *G* = 1 means that all the TCR clones are the same. When the variation of TCR repertoire is large, *G* approaches 0. The raw data supporting the conclusions of this manuscript, except the private information of the subjects, will be made available by the authors, without undue reservation, to any qualified researchers.

### Statistical analyses

Statistical analyses were performed with the JMP Pro 13.0.0 statistical analysis program (SAS Institute Inc., USA) and SPSS version 23 software (IBM, USA). The statistical tests used to determine statistical significance are indicated in the respective figure legends. Continuous variables are presented as median values with interquartile range, unless otherwise specified. A two-tailed *p* < 0.05 was considered significant.

## Results

### Clinical characteristics

Clinical characteristics of the subjects are shown in Table 1. Maternal ages of 1st trimester miscarriage and 3rd trimester normal pregnancy were higher than that of 1st trimester normal pregnancy. The frequency of cesarean section showed no significant difference between 3rd trimester normal pregnancy and preeclampsia.

**Table 1:** Demographic and clinical characteristics.

	**1st trimester normal pregnancy**	**3rd trimester normal pregnancy**	**1st trimester miscarriage with abnormal embryo**	**1st trimester miscarriage with normal embryo**	**3rd trimester preeclampsia**
	**(*****n*** = **9)**	**(*****n*** = **12)**	**(*****n*** = **11)**	**(*****n*** = **7)**	**(*****n*** = **7)**
Maternal age (years), median (IQR)	28 (24–31)	36 (33–38)^†^	39 (38–41)^†^	38 (32–40)^†^	38 (31–41)
Gravidities, median (IQR)	3 (1–4)	5 (2–5)	3 (2–4)	3 (2–4)	2 (1–4)
No. of liveborn children, median (IQR)	0 (0–2)	0 (0–1)	0 (0–0)	0 (0–1)	0 (0–1)
No. of miscarriages, median (IQR)	0 (0–2)	2 (0–3)	1 (0–2)	1 (1–3)	(0–2)
Past history of stillbirth, *n* (%)	0 (0.0)	3 (25.0)	1 (9.1)	1 (14.3)	0 (0.0)
Nullipara, *n* (%)	5 (55.5)	5 (41.7)	8 (72.7)	3 (42.9)	4 (57.1)
Gestational weeks, median (IQR)	7 (7–9)	39 (38–40)	9 (8–10)	8 (8–10)	34 (32–37)
Cesarean section, *n* (%)		4 (33.3)			5 (71.4)

*IQR, interquartile range. Steel–Dwass test for continuous variables and Fisher's exact test for categorical variables*.

† *p < 0.05 vs. 1st trimester normal pregnancy*.

### TCR repertoire analysis of effector treg cells in normal pregnancy

**Figure 2:**
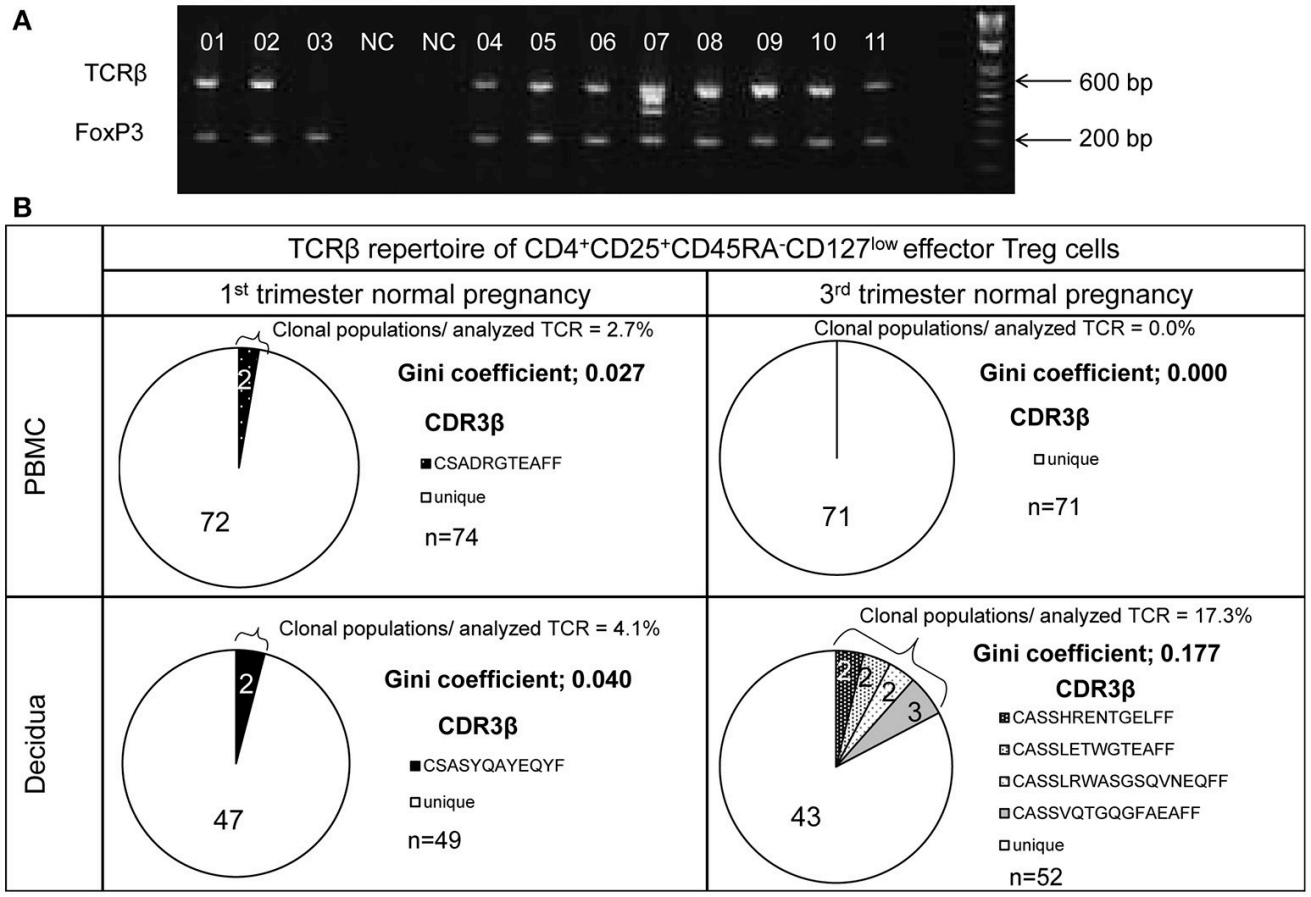
TCRβ repertoire analysis of CD4^+^CD45RA^−^CD25^+^CD127^low/−^ effector Treg cells. **(A)** TCRβ and FoxP3 cDNAs amplified by multiplex one-step RT-PCR were resolved by agarose gel electrophoresis. Each single cell was numbered. NC denotes the cell-free negative control. FoxP3 and TCRβ mRNAs were expressed in 11/11 and 10/11, respectively. **(B)** Representative data of the TCRβ repertoire of CD4^+^CD45RA^−^CD25^+^CD127^low/−^effector Treg cells in PBMC and decidua. The TCR repertoires were determined by amino acid and nucleotide sequences of complementarity determining region 3 (CDR3) of TCRβ chain. Each pie chart slice (shaded or closed) indicates clonal T cell population with the same clonotypic TCRβ. An open pie chart slice indicates T cells with unique TCRβ. Frequency of clonal populations among analyzed TCRβ and the Gini coefficients of the TCRβ repertoire were calculated.

TCRβ and FoxP3 cDNAs were amplified from single cells and electrophoresed in agarose gel (Figure 2A). The number of analyzed TCRβ sequences of decidual effector Treg cells in 1st trimester normal pregnancy, 3rd trimester normal pregnancy, 1st trimester miscarriage with abnormal or normal embryo, and 3rd trimester pregnancy with preeclampsia for each subject was 49 (35.5–60), 46 (32.3–77.3), 36 (29–56), 43 (27–53), and 42 (31–45), respectively. The number of analyzed TCRβ sequences of effector Treg cells in PBMC in 1st trimester and 3rd trimester normal pregnancies was 70 (61.5–73.5) and 52 (43.5–60.8), respectively. Representative TCRβ repertoires in 1st trimester and 3rd trimester normal pregnancies are shown in Figure 2B. In the 3rd trimester, decidual effector Treg cells were clonally expanded and TCR repertoires were skewed (Figure 2B).

**Figure 3:**
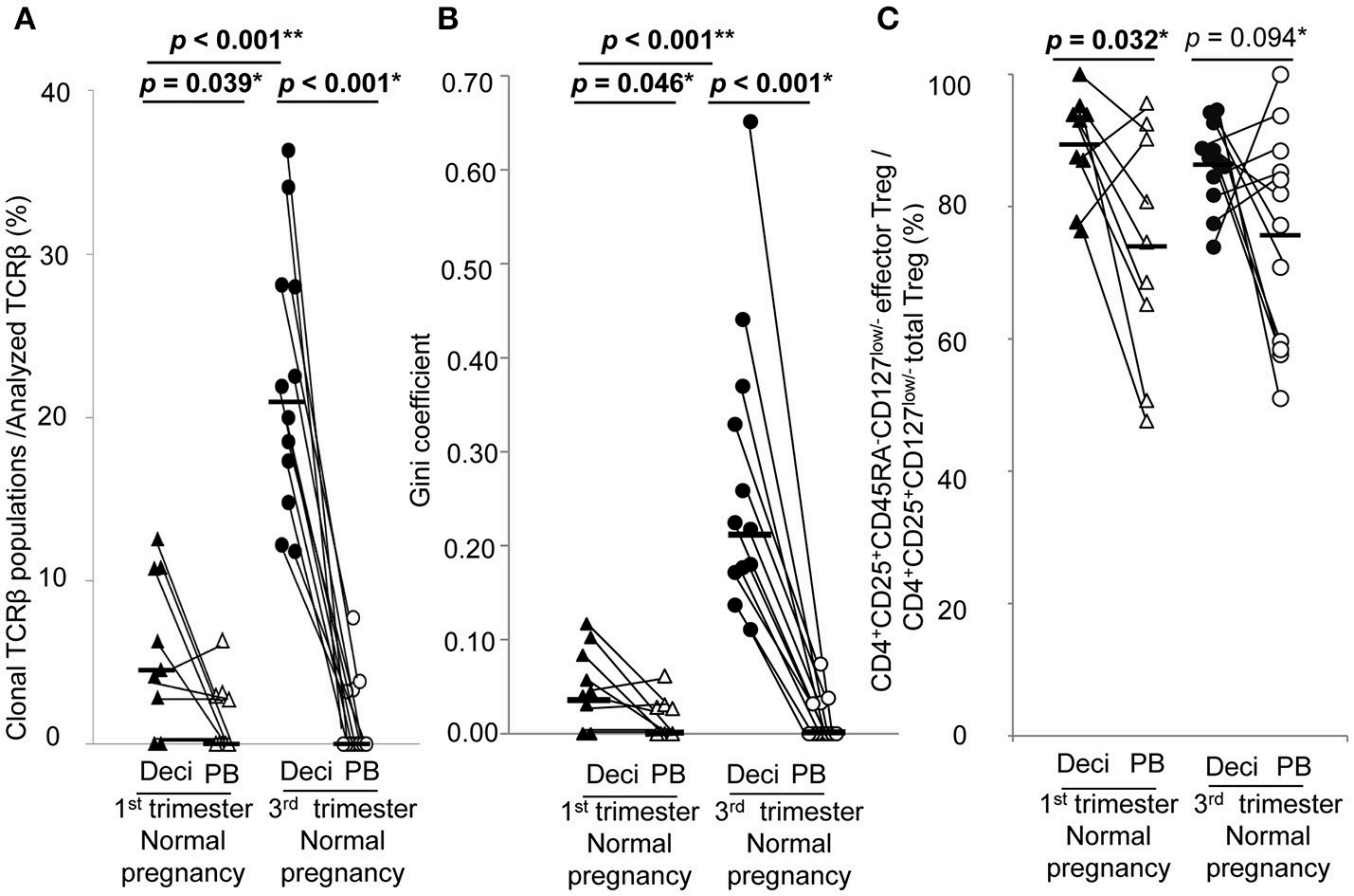
TCRβ repertoire and flow cytometric analysis of effector Treg cells in 1st and 3rd trimester decidua and peripheral blood in normal pregnancy. **(A)** Frequencies of clonal populations among the analyzed TCRβ of effector Treg cells in 1st (*n* = 9) and 3rd trimester (*n* = 12) decidua and peripheral blood in normal pregnancies. **(B)** Gini coefficient of TCRβ repertoire of effector Treg cells. **(C)** Ratio of CD4^+^CD45RA^−^CD25^+^CD127^low/−^ effector Treg cells per CD4^+^CD25^+^CD127^low/−^ total Treg cells. *p from Wilcoxon signed-rank test and **p from Mann–Whitney *U*-test. Each dot represents one donor, lines indicate median. Deci, decidua; PB, peripheral blood.

In normal pregnancy, the ratio of clonal populations of decidual effector Treg cells in the 3rd trimester was increased compared with the 1st trimester [4.5% (1.4–10.8%) vs. 20.9% (15.4–28.1%), *p* < 0.001]. In peripheral blood, the ratio for clonal populations of effector Treg cells was significantly smaller than that in paired decidual samples [0.0% (0.0–3.0%) vs. 4.5% (1.4–10.8%), *p* = 0.039 in the 1st trimester, 0.0% (0.0–3.3%) vs. 20.9% (15.4–28.1%), *p* < 0.001 in the 3rd trimester] and was not increased even in the 3rd trimester [0.0% (0.0–3.0%) vs. 0.0% (0.0–3.3%), *p* = 0.935; Figure 3A]. The Gini coefficient of the decidual TCRβ repertoire of effector Treg cells was higher in the 3rd trimester than in the 1st trimester [0.04 (0.02–0.09) vs. 0.22 (0.17–0.36), *p* < 0.001]. The Gini coefficient of the TCRβ repertoire of effector Treg cells in PBMC was lower than that in paired decidual samples in the 1st and 3rd trimesters [0.00 (0.00–0.03) vs. 0.04 (0.02–0.09), *p* = 0.046 in the 1st trimester, 0.00 (0.00–0.04) vs. 0.22 (0.17–0.36), *p* < 0.001 in the 3rd trimester; Figure 3B].

Frequencies of effector Treg cells among total Treg cells of 1st trimester decidua were significantly higher than that of peripheral blood [93.0% (82.4–94.6%) vs. 74.6% (58.0–91.4%), *p* = 0.032]. In the 3rd trimester, the *p*-value did not reach a significant level [87.0% (82.4–91.7%) vs. 79.6% (58.7–87.6%), *p* = 0.094; Figure 3C].

**Figure 4:**
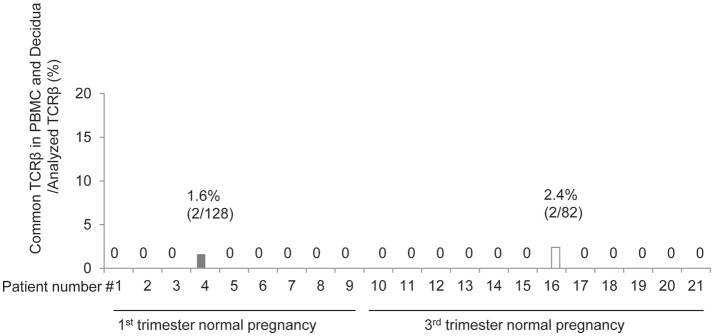
Common clonotypic effector Treg cells between PBMC and decidua in 1st and 3rd trimester decidua and peripheral blood in normal pregnancy. Frequencies of common TCRβ in PBMC and decidua among analyzed TCRβ of effector Treg cells.

Next, we compared the TCR repertoires of peripheral blood and decidual effector Treg cells in each case. Common clonotype of effector Treg cells between PBMC and decidua appeared only in case #4 (two clones among 128 clones; 56 from decidua and 72 from PBMC) and case #16 (two clones among 82 clones; 40 from decidua and 42 from PBMC). The findings suggested marked differences in the characteristics of Treg cells between peripheral blood and decidua (Figure 4).

### TCR repertoire of decidual effector treg cells between previous and subsequent pregnancy

**Figure 5:**
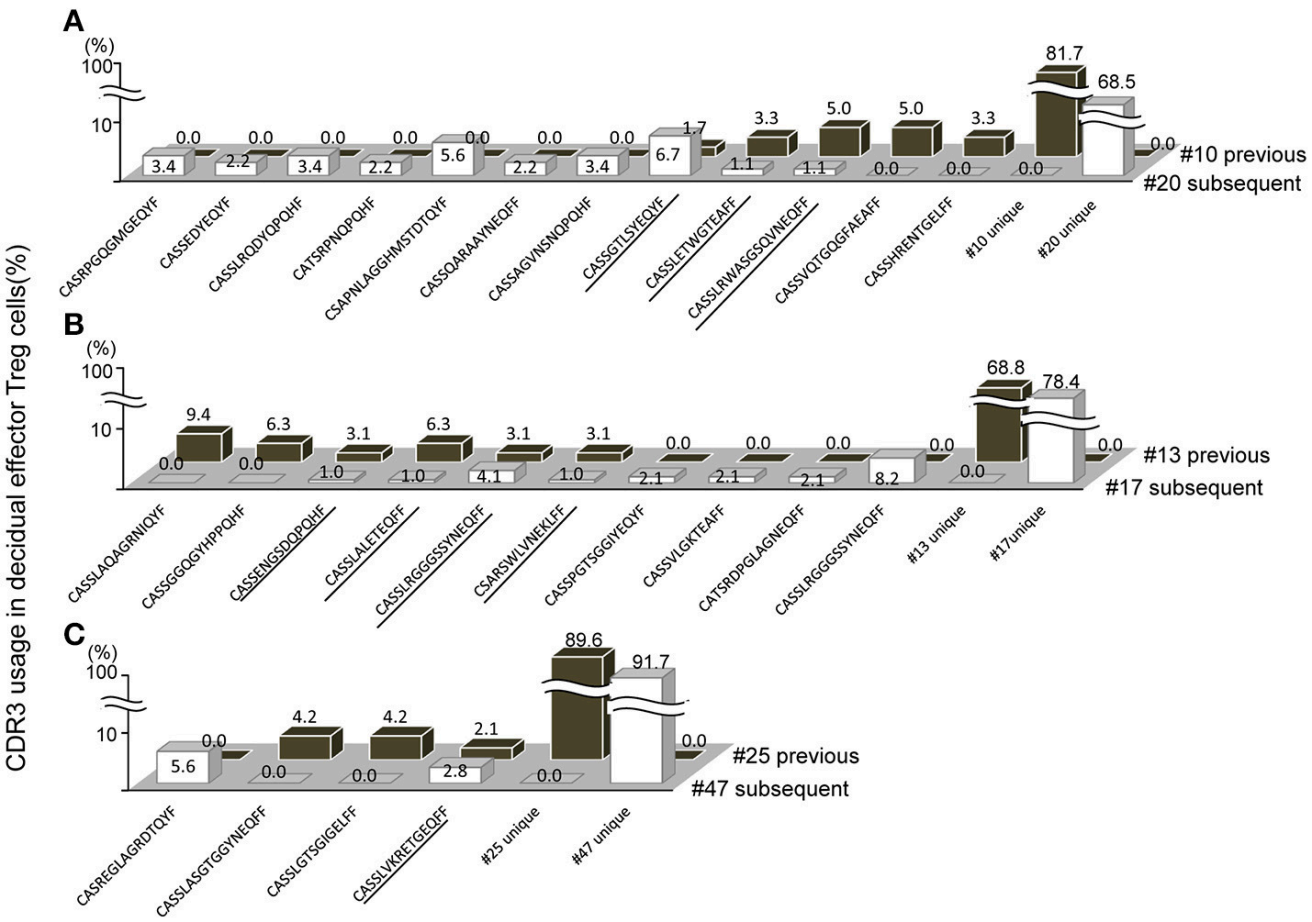
Shared TCRβ repertoire of decidual effector Treg cells between previous and subsequent pregnancy. **(A–C)** Frequencies of decidual effector Treg cell clones expressing clonotypic TCRβ with the indicated CDR3 amino acid sequence. The underlined CDR3 sequences were shared in previous and subsequent pregnancies. **(A)** Case #10 previous pregnancy and case #20 subsequent pregnancy ending in the 3rd trimester with a normal vaginal delivery. **(B)** Case #13 previous pregnancy and case #17 subsequent pregnancy ending in the 3rd trimester with a normal vaginal delivery. **(C)** Case #25 previous pregnancy ending in miscarriage with a normal embryo and case #47 subsequent pregnancy ending in miscarriage with an abnormal embryo.

When repertoires of effector Treg cells were compared between past and subsequent pregnancies of the same subjects, sharing of some part of the repertoire of decidual effector Treg cells was evident. In case A, the TCR repertoires of cases #10 and #20, a pair of previous and subsequent 3rd trimester normal vaginal deliveries, shared three clones among 149 clones (Figure 5A; shared clones are underlined). In case B, the TCR repertoires of cases #13 and #17 revealed four shared clones among 129 clones (Figure 5B; underlined). In case C, case #25 was a previous pregnancy and resulted in miscarriage with normal embryo and case #47 was a subsequent pregnancy of the same subject and resulted in miscarriage with abnormal embryo. This pair shared one clone among 84 clones (Figure 5C; underlined). None of the TCR repertoires of effector Treg cells in PBMC were shared between previous and subsequent pregnancies (data not shown). Among the subjects who experienced pregnancies several times during the study period, no subject had their pregnancies ending up in preeclampsia.

### TCR repertoire of decidual effector treg cells of normal pregnancy and miscarriage

**Figure 6:**
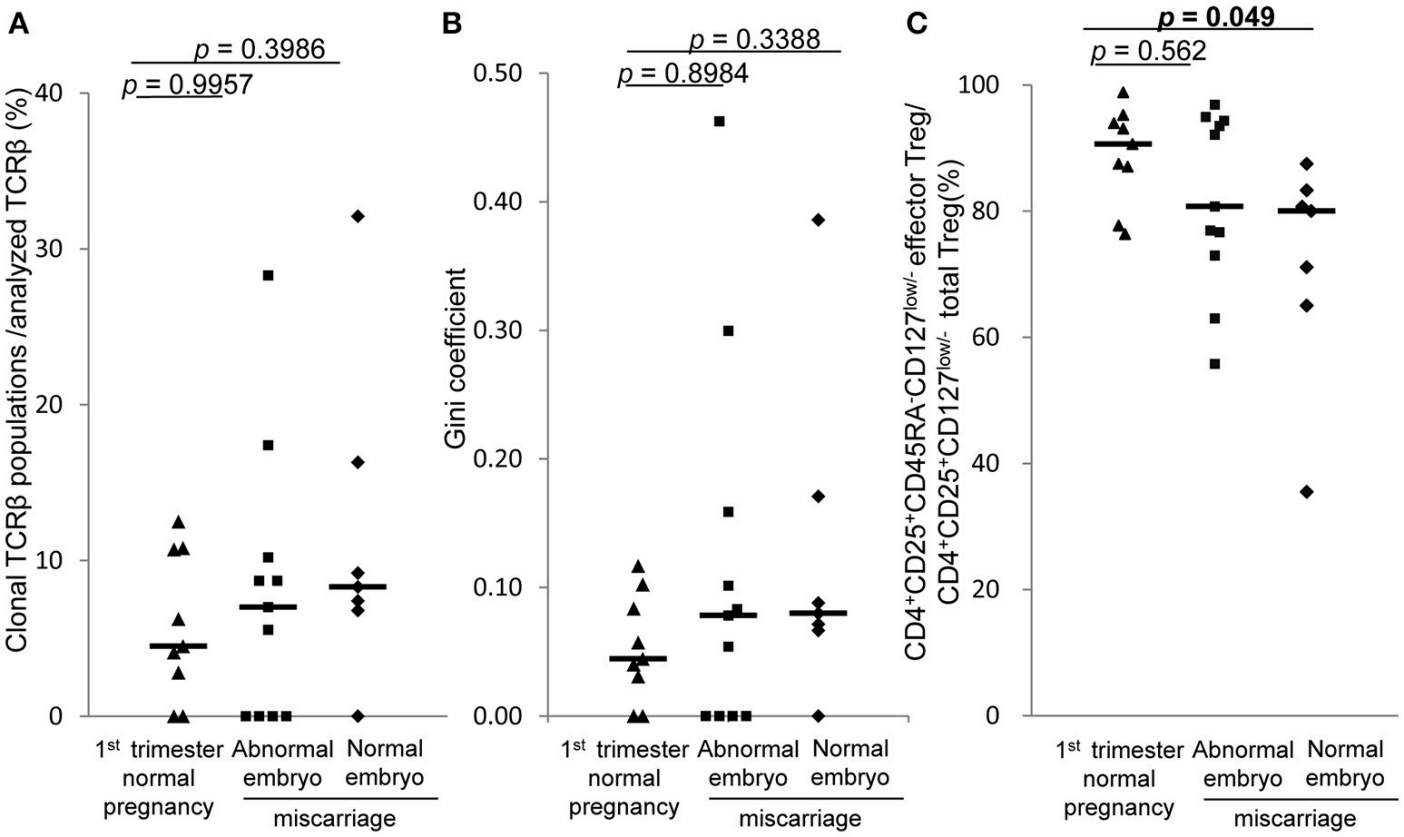
TCRβ repertoire and flow cytometric analysis of decidual effector Treg cells in 1st trimester normal pregnancy and miscarriage. **(A)** Frequencies of clonal populations among analyzed TCRβ of effector Treg cells in 1st trimester normal pregnancy (*n* = 9) and miscarriage with abnormal embryo (*n* = 11) or normal embryo (*n* = 7). **(B)** Gini coefficient of TCRβ repertoire of effector Treg cells. **(C)** Ratio of CD4^+^CD45RA^−^CD25^+^CD127^low/−^ effector Treg cells per CD4^+^CD25^+^CD127^low/−^ total Treg cells. *p from Steel's test. Each dot represents one donor; lines indicate median.

In 1st trimester decidua, the frequencies of clonal populations of effector Treg cells and the Gini coefficients of TCR repertoires of miscarriage with abnormal embryo showed no significant differences between normal pregnancy, miscarriage with abnormal embryo, and miscarriage with normal embryo (Figures 6A,B). Proportions of CD4^+^CD45RA^−^CD25^+^CD127^low/−^ effector Treg cells among CD4^+^CD25^+^CD127^low/−^ total Treg cells in decidua were significantly lower in miscarriage with normal embryo than normal pregnancy [80.0% (65.0–83.3%) vs. 90.6% (82.3–94.6%), *p* = 0.049; Figure 6C].

### TCR repertoire of decidual effector treg cells of normal pregnancy and preeclampsia in 3rd trimester

**Figure 7:**
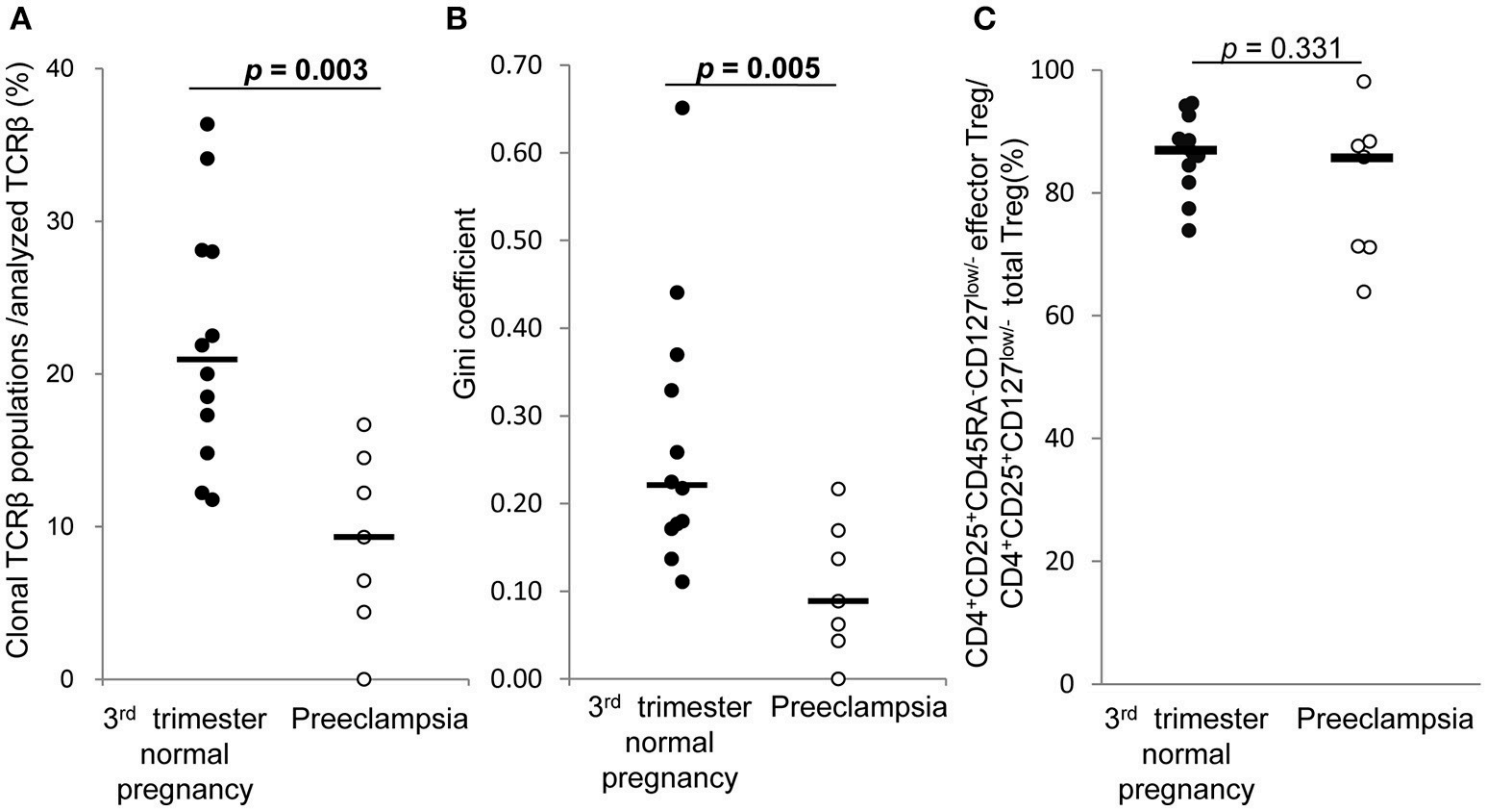
TCRβ repertoire and flow cytometric analysis of decidual effector Treg cells in 3rd trimester normal pregnancy and preeclampsia. **(A)** Frequencies of clonal populations among analyzed TCRβ of effector Treg cells in 3rd trimester normal pregnancy (*n* = 12) and preeclampsia (*n* = 7). **(B)** Gini coefficient of TCRβ repertoire of effector Treg cells. **(C)** Ratio of CD4^+^CD45RA^−^CD25^+^CD127^low/−^ effector Treg cells per CD4^+^CD25^+^CD127^low/−^ total Treg cells are shown. *p from Mann–Whitney *U*-test. Each dot represents one donor; lines indicate median.

In 3rd trimester decidua, frequencies of clonal populations of effector Treg cells (Figure 7A) and Gini coefficients of TCR repertoires (Figure 7B) were significantly lower in preeclampsia than normal pregnancy [9.3% (4.4–14.5%) vs. 20.9% (15.4–28.1%), *p* = 0.003 and 0.09 (0.04–0.17) vs. 0.22 (0.17–0.36), *p* = 0.005, respectively]. The proportions of CD4^+^CD45RA^−^CD25+CD127^low/−^ decidual effector Treg cells among CD4^+^CD25+CD127^low/−^ decidual total Treg cells did not differ significantly between the two groups [85.8% (71.1–88.4%) vs. 87.0% (82.4–91.7%), *p* = 0.331] (Figure 7C).

## Discussion

This study is the first report of the increase of clonally expanded decidual effector Treg cells in late gestation of normal pregnancy. In contrast, the repertoire of effector Treg cells of PBMC was not skewed in the 3rd trimester. A previous study showed that TRBV repertoires of total Treg cells differ in PBMC and decidua in the 3rd trimester (22). Our data support this finding. Neller et al. used 25 TRBV monoclonal antibodies in a TCR repertoire analysis. The analysis could not determine whether the skewed TCR repertoire occurred due to clonal expansion or not. We analyzed TCR repertoires more precisely based on CDR3 sequences that are a part of antigen binding site and provide high variety of TCR. Our results reveal that the skewed TCR repertoire in effector Treg cells in decidua, but not in peripheral blood during pregnancy, reflects clonal expansion of effector Treg cells in the decidua.

Concerning the relationship between the maldistribution or dysfunction of Treg cells and pregnancy complications, such as recurrent pregnancy loss or preeclampsia, the failure to maintain feto-maternal tolerance is thought to be one of the causes of these diseases (16–21). In the 1st trimester, miscarriage with normal embryo showed decreased population of decidual effector Treg cells compared with normal pregnancy. This result is consistent with our previous report of the decreased proportion of effector Treg cells in miscarriage with normal embryo than 1st trimester normal pregnancy (15). In contrast, the TCR repertoire in this study showed no significant skew between these populations (Figure 6). Taken together, our findings suggest that the decreased number of decidual effector Treg cells might be related to the pathogenesis of miscarriage with normal fetal karyotype, rather than an altered TCR repertoire. On the other hand, preeclampsia showed insufficient clonal expansion of decidual effector Treg cells compared with normal pregnancy in the 3rd trimester. In the 3rd trimester, effector Treg cells are the most dominant subset among Treg cells in PBMC and at the uteroplacental interface (14). The decrease in clonal populations of decidual effector Treg cells might be related to the pathogenesis of preeclampsia.

Concerning the TCR clonotypes of decidual effector Treg cells, we show for the first time that some TCR clonotypes in decidual effector Treg cells are shared between previous and subsequent pregnancies of the same subjects, but not those in PBMC. The lower limit for the number of different CDR3 amino acid sequences in TCRβ is estimated to be ~2 × 10^7^ in young humans (28). This indicates that there is a very low probability of coincidence of the CDR3 amino acid sequence between two independent Treg cells if there is no force to skew the populations. Thus, effector Treg cell clones shared between previous and subsequent pregnancies might be recruited by reacting to the same antigens expressed in feto-maternal interface, suggesting that fetal antigen-specific Treg cells might accumulate at the feto-maternal interface.

The existence of fetal antigen-specific Treg cells and those recruited to the feto-maternal interface during pregnancy were reported in mice (8–10). An examination of functional differences of Treg cells from PBMC and decidua in humans led to the suggestion that decidual Treg cells contain fetal-antigen specific populations (11). However, fetal antigen-specific Treg cell clones have not been identified in humans. The present finding of common Treg cell clones in the decidua in previous and subsequent pregnancies raises the possibility that these clones might react to antigens expressed in feto-maternal interface and, thus, could be candidates of fetal antigen-specific Treg cells. Regarding the target antigen presenting cells for Treg cells in human decidua, it has been reported that HLA-C mismatched pregnancies feature an increased amount of Treg cells and higher activation of conventional T cells than non-mismatched pregnancies (29). HLA-C, E, F, and G are expressed in extravillous trophoblasts (30–33). These expressed HLAs might be potent antigens recognized by Treg cells.

Some limitations of our study have to be considered. Firstly, we could not amplify TCRα as efficiently as TCRβ. Thus, the TCR repertoire was analyzed based on TCRβ and not paired TCR. Secondly, our single cell TCR repertoire analysis covered only a limited number of Treg cell clones compared with the bulk TCR repertoire analysis by next-generation sequencing. Thirdly, the functionality of TCRs was not assessed. TCRs derived from antigen-specific Treg cells and their function were demonstrated by using TCR^mini^ mice, whose TCRβ is identical in all T cells (34, 35). In humans, a functional assay of target-specific polyclonal Treg cells has been developed (27). However, a direct functional assay of TCRs obtained from Treg cells is still not available, reflecting the limited knowledge about epitopes from physiological targets of Treg cells (27, 36). TCRs from tumor specific conventional CD4^+^ T cells and CD8^+^ T cells were identified using single-cell RT-PCR methods (23, 24, 37, 38). We also demonstrated a rapid and efficient functional assay for TCRs directly obtained from CD8^+^ T cells using TCR-transduced lymphocytes in the absence of information on antigen or MHC haplotype (38). We tried to apply this technique to mixed lymphocyte reaction of paired αβ TCR-transduced maternal T cells or TCR-negative T cell lines against self-fetal umbilical cord blood but were unsuccessful. An assay method capable of identifying target-specific Treg or CD4^+^ T cells using TCR-transduced responder cells in the absence of information concerning their antigen or MHC haplotype, to the best of our knowledge, has not been reported yet. It might be technically more difficult than analysis of TCRs obtained from CD8^+^T cells, thus novel strategy might be needed to improve assay method of TCRs obtained from CD4^+^ T cells. When the assay method is established, the function of obtained TCRs will be explored. Fourthly, we have not been able to demonstrate a relationship between the TCR clonotype and effector molecule expression. Zemmour et al. showed that Treg cells, which share the same TCR clonotype, display similar transcriptional identities by single-cell analysis in mice (39). Combined single-cell TCR and gene expression analysis should be addressed, thereby providing further insight into features of heterogeneous decidual Treg cells.

In summary, we show for the first time that effector Treg cells are clonally expanded in 3rd trimester decidua, but not in peripheral blood in humans. In preeclampsia, the TCR repertoires of decidual effector Treg cells were not skewed. Clonally expanded effector Treg cell populations might be more important in the 3rd trimester than in the 1st trimester. Insufficient expansion of clonal effector Treg cells in decidua might be an etiological aspect in the development of preeclampsia. On the other hand, the frequency of effector Treg cells among the total Treg cells decreased in cases of miscarriage with normal fetal chromosomal content, but clonal effector Treg cells did not decrease. Our findings further the understanding of the mechanisms of feto-maternal tolerance and could provide clues for understanding the different aspects of the pathophysiology of preeclampsia and miscarriage.

## Ethics statement

This study was carried out in accordance with the recommendations of Ethical Guidelines for Medical and Health Research Involving Human Subjects, the Ministry of Health, Labor and Welfare, Japan. The protocol was approved by the ethics review committee of University of Toyama (Rin 28-144). All subjects gave written informed consent in accordance with the Declaration of Helsinki.

## Author contributions

SS, HK, and AM: conception and design; ST, XZ, and AU: acquiring and processing samples; ST, XZ, HK, HH, AU, and KT: execution of experiment; ST and XZ: analysis of data; ST, XZ, HK, HH, TS, and SS: interpretation of data; ST, TS, and SS: drafting manuscript; XZ, HK, HH, TS, AU, KT, and AM: revision of the manuscript for important intellectual content.

### Conflict of interest statement

The authors declare that the research was conducted in the absence of any commercial or financial relationships that could be construed as a potential conflict of interest.

## Supplementary material

Supplementary Table 1 Primers for single-cell RT-PCR.

Supplementary Table 2 Contents of single-cell RT-PCR reaction mix.
